# The COVID‐19 pandemic and healthcare workers psychological well‐being: a cross‐sectional survey in Indonesia

**DOI:** 10.1002/nop2.1034

**Published:** 2021-08-24

**Authors:** Ni’matuzahroh, Valendriyani Ningrum, Widayat, Myrtati Dyah Artaria, Mein‐Woei Suen

**Affiliations:** ^1^ Department of Psychology Asia University Taiwan; ^2^ Department of Healthcare Administration Specialty in Psychology Asia University Taiwan; ^3^ Faculty of Psychology University of Muhammadiyah Malang Indonesia; ^4^ Department of Dental Public Health Faculty of Dentistry Baiturrahmah University Indonesia; ^5^ Department Management Faculty of Economics and Business University of Muhammadiyah Malang Indonesia; ^6^ Department of Anthropology Faculty of Social and Political Sciences Universitas Airlangga Indonesia; ^7^ Gender Equality Education and Research Center Asia University Taichung Taiwan; ^8^ Department of Medical Research Asia University Hospital, Asia University Taiwan; ^9^ Department of Medical Research China Medical University Hospital, China Medical University Taiwan

**Keywords:** COVID‐19 pandemic, healthcare workers, psychological well‐being

## Abstract

**Aim:**

This study was conducted to investigate the relationships amongst psychological well‐being (PWB), emotional intelligence and coping strategies.

**Design:**

This study employed a cross‐sectional survey design.

**Method:**

A total of 146 healthcare workers (HCWs) were enrolled in this study. They were asked to finish several questionnaires, including the Wong and Law Emotional Intelligence Scale, the Brief‐Coping Orientation to Problems Experienced Scale and the PWB Scale. The obtained data were analysed using partial least squares structural equation modelling, employee SmartPLS, to estimate the contributions of influencing factors and evaluate the moderating effect of coping strategy (CS) on the relationship between emotional intelligence and PWB.

**Results:**

Results revealed that emotional intelligence influenced PWB, and CS moderated both emotional intelligence and PWB. Furthermore, CS plays an essential role in improving PWB related to emotional intelligence amongst HCWs during the COVID‐19 outbreak.

## 1 INTRODUCTION

The COVID‐19 virus, first detected in Wuhan, China, on 31 December 2019, continues to affect many countries. In fact, at present, the virus is said to have mutated into a new, more dangerous variant. Thousands of people throughout the world have died as a result of this pandemic (Du et al., 2020). A prolonged pandemic influences various aspects of human life, and not a single party can predict when it will stop. In the wake of this phenomenon, the most affected sectors are business industries engaged in visually identifiable goods and services (Carnevale & Hatak, 2020). Another industry that has been significantly affected is the health services sector, including the medical sector, specialized clinics and hospitals that serve different polys. As a result of the virus outbreak, hospitals and health facilities have been required to participate and offer non‐stop services related to the treatment of symptomatic COVID‐19 patients.

For many months now, many hospitals or health facilities have experienced an influx of COVID‐19 patients, and such an increase has affected healthcare workers (HCWs) in various ways. First, HCWs have had to work overtime and face high risks of infection due to long‐term, direct contact with patients (Burdorf et al., 2020; McDougall et al., 2021b). Second, they also suffer from social conflict and psychological well‐being (PWB) problems resulting from their emotional involvement with some of their patients. This kind of involvement entails dealing with other possible circumstances, such as sadness, fear, harassment or neglect of their work (Ertem et al., 2020; Leiter & Maslach, et al. 2009).

Cao et al. (2020) reported that, since the COVID‐19 situation broke out, many HCWs have experienced various symptoms of psychological disorders, such as low appetite, sleep disturbance, negative emotions (including worry and loss of family members), fear of infection, stress due to heavy workloads and bodily discomfort. This finding is in line with several elements of well‐being presented in the scientific literature. Related to these, the most common causes for the abandonment of positions amongst HCWs include poor salaries, too many obligations, health issues, psychosocial burdens and burnout (Dall'Ora et al., 2015; Li et al., 2011). Similarly, Kowalczuk et al. (2020) confirmed that working excessively and burnout are pivotal events affecting employees' mental well‐being. Excessive workloads lead to burnout symptoms, thus highlighting the need for healthcare professionals to be given breaks more often.

In contrast, Chen et al. (2020) found that even though HCWs displayed excitement, irritability, lack of rest and signs of psychological distress, they often denied any psychological assistance and claimed to not have any problems. This suggests that HCWs may lack emotional intelligence and effective coping strategies, which are considered essential in dealing with the uncertainties of the COVID‐19 situation and the resulting workload they experience (Aldao et al., 2010; Gross & John, 2003; Moore et al., 2008; Rantanen et al., 2011; Webb et al., 2012). In particular, both factors determine PWB. Montes‐berges (2007) reported that nurses who had high emotional intelligence had better mental well‐being than those with low emotional intelligence. To the best of our knowledge, this is the first study of its kind to examine such a topic.

Emotional intelligence endows a person the abilities to consider and acknowledge another person's feelings, to set objectives to improve one's potential and to see experiences from other perspectives (Gerits et al., 2005). Khalid et al. (2016) found that HCWs who use ineffective coping are more likely to experience stress that can impair their PWB compared to those who use positive coping. Gerits et al. (2005) found that nurses with higher problem‐solving and stress‐tolerant abilities often reported less burnout. We argue that this topic requires urgent research topic given that, according to Bawalsah (2016), improving the PWB of HCWs is a vital component of exercising vigilance and effectively fighting against emerging infectious diseases.

Thus, we decided to investigate the relationships between the PWB of HCWs and their emotional intelligence and coping strategies during the COVID‐19 pandemic. As mentioned earlier, this is an important topic to explore, because improving HCWs’ PWB is crucial in maintaining efficacy and public health during the pandemic.

## 2 BACKGROUND

PWB is commonly conceived as the mental capacity to solve problems, adapt to the environment and meet output targets (Altaras Dimitrijević et al., 2018). Meanwhile, Keyes et al. (2002) defined “psychological well‐being” by incorporating Maslow's six‐dimensional roles of PWB with the ideas of Rogers, Jung and Ericson and coming up with the following dimensions: self‐acceptance (positive attitude towards self), positive relationships with others, autonomy, environmental mastery, purpose in life and personal development.

Philip and Cherian (2020) reported that the factors influencing HCWs’ PWB during an outbreak include poor coping strategies, insufficient social support, increased patient interactions, challenging work conditions, inadequate preparation, quarantine, high perceived risks, stigma, social isolation, lack of resilience and a history of physical or mental health problems. Strong evidence has also been shown proving that emotional intelligence capability predicts facets of PWB and the positive relationship between life satisfaction and subjective happiness (Guerra‐Bustamante et al., 2019).

In comparison, “emotional intelligence” refers to the ability to interpret, appraise and articulate emotions accurately; the ability to access and produce feelings as they facilitate thinking; the ability to recognize emotions and emotional knowledge; and the ability to control emotions to encourage emotional and intellectual development (Wong & Law, 2002). Austin et al. (2005) and Parker et al. (2004) showed that emotional intelligence is an important variable that determines the success of medical students.

Meanwhile, studies have found that regulating emotions influences individuals’ well‐being (Gross & John, 2003) and higher involvement with their work (Barreiro & Treglown, 2020). Furthermore, it has been shown that a person's failure to regulate emotional reactions to daily experiences and choose appropriate behavioural reactions to manage emotions both have an impact on several outcomes, including interpersonal relationships, physical health complaints, behavioural problems and decreased levels of resilience/survival (Aldao et al., 2010; Gross & John, 2003; Moore et al., 2008; Tamir et al., 2020).

Normally, HCWs are expected to have high levels of emotional intelligence to help them cope with emergency situations, to react appropriately to certain feelings and to empathize with the patients who are facing various challenges under their care. Related to this, Gerits et al. (2005) showed that nurses with high emotional intelligence typically showed minor signs of burnout, the least absenteeism due to sickness and the most negligible work turnover rates. Similarly, Gustems‐Carnicer et al. (2019) provided support to previous research by stating that emotional intelligence increases happiness and emotional well‐being.

A coping strategy (CS) refers to a person's effort to prevent, minimize or control stress (Rantanen et al., 2011) and is a variable that affects a person's PWB (Landen & Wang, 2010). Coping is an effort to prevent anything terrible from happening in one's life (Rantanen et al., 2011) and is a means of self‐balancing by reducing stress or negative emotions Bonab et al. (2017). A study has shown that someone who uses effective coping can better manage stress compared to those who use ineffective coping, which can be harmful to their PWB (Costa et al., 2015). A study by Dall'Ora et al. (2015) found that nurses with higher problem‐solving and stress‐tolerant abilities often reported less burnout compared to other nurses who did not have the same abilities. On the basis of the above discussion, we propose the following hypotheses:

Hypothesis 1: Emotional intelligence is positively correlated to PWB.

Hypothesis2: CS moderates the relationship between emotional intelligence and PWB.

This study's findings can help HCWs recognize these effects and provide basic information on how to develop nursing strategies to address HCWs’ PWB problems.

### 2.1 Research questions

This study attempts to address the following research questions:

- What is the correlation between emotional intelligence and PWB of HCWs during the COVID‐19 pandemic?
- How does CS play a moderating role in the relationship between emotional intelligence and HCWs’ PWB during the COVID‐19 pandemic?

## 3 METHODS

### 3.1 Design and participants

This cross‐sectional research was conducted on HCWs (medical, nursing and volunteer COVID‐19 workers) who worked in hospitals or clinic centres in the province from July–November 2020. The participants were sourced from the registered participant pool using a Google online survey form. The age range was divided into three age groups (20–34, 35–50, and >50 years). A total of 146 HCWs from East Java, Indonesia, participated in the survey. A survey form was commissioned to finalize an online survey using a questionnaire. First, the participants were contacted by social media. Next, the contributors were randomly selected, after which a link was sent inviting them to join the study. The participants were informed that their involvement was voluntary and confidential. Upon submitting a signed letter of consent, they were then given the questionnaire on Google Forms and were asked to fill it. The researchers were blind to the identities of the selected participants.

### 3.2 Measures

#### 3.2.1 Demographic information questionnaire

The participants completed a questionnaire extracting their contact and demographic information, including their gender, age and educational level.

#### 3.2.2 Wong and Law emotional intelligence scale (WLEIS)

This study used the WLEIS developed by Wong and Law (2002) to measure emotional intelligence. The WLEIS is a 16‐item self‐report trait of emotional intelligence, which is determined using a 5‐point Likert scale (1 = totally disagree, 5 = totally agree). The measure consists of four correlated scales consisting of four elements each: self‐emotion assessment, other emotion assessment, use of emotion and emotion control. Internal consistency reliability for the four factors (each with four items) in the study of Wong and Law (2002) ranged from 0.83–0.90, whilst the confirmatory factor analysis shows a reasonably good fit. The model c2 was 591.59 (*df* = 398), the standardized root mean square residual was .08, the comparative fit index was .90, and the Tucker–Lewis index was .89. These results indicate good convergent and discriminant validities between our emotional intelligence (EI) and the Big Five personality dimensions.

In the current sample, the total score was used to calculate internal consistency. The excellent internal consistency (α = .091) and composite reliability (.93) illustrate that the construction of latent variables in the measurement model is reliable.

#### 3.2.3 The brief‐coping orientation to problems experienced (COPE) scale

The current study used the Brief‐COPE Scale (Carver, 1997) to measure CS. This scale comprises 28 questions (two question items in each strategy) and assesses 14 stress‐coping strategies. A 4‐point Likert scale answers each question in which score of 1–4 is given for each question, and a score ranging from 2–8 is given for each strategy. The participants rate the items as they assess whether they have been using each way of coping. The internal consistency of the COPE scale reported by Carver (1997) was generally high (.62), and the test–retest suggests that self‐reports of coping styles are relatively stable. In a study by Lai et al. (2015), the internal consistency evaluations of the Brief‐COPE total and subscale scores were adequate, with Cronbach's α coefficient values of .92 (Brief‐COPE total), .76 (Active Avoidance coping), .89 (problem‐focussed coping), .83 (positive coping) and .69 (religious/denial coping).

In this study, CS as a latent variable consisted of valid measuring items. The total score showing good internal consistency (α = .87) illustrates that the measurement model is valid. Meanwhile, the composite reliability of .90 indicates that the construction of latent variables in the measurement model is reliable.

#### 3.2.4 Psychological well‐being scale

PWB was measured in this study using the PWB scale from Ryff (1989). This scale measures six subscales covering the PWB definition: self‐acceptance, environmental mastery, personal development, positive relationships with others, goals and autonomy in life. This is an area of 20 elements that are divided almost equally between positive and negative elements. This subscale has the following satisfactory internal consistency: self‐acceptance, .93; positive relations with others, .91; autonomy, .86; environmental mastery, .90; purpose in life, .90; and personal growth, .87. The test–retest reliability coefficients for the 20‐item scales over six weeks on a subsample of respondents (*N* = 117) were as follows: self‐acceptance, .85; positive relations with others, .83; autonomy, .88; environmental mastery, .81; purpose in life, .82; and personal growth, .81.

In the present study, the total score was used to calculate internal consistency, which showed good internal consistency (α = .066). The composite reliability is greater than .70, illustrating that the construction of latent variables in the measurement model is reliable.

### 3.3 Data analyses

The data were analysed using partial least squares structural equation modelling (PLS‐SEM), employee SmartPLS. The researchers used descriptive reports, including means and standard deviations for continuous data, demographic data frequencies and proportions. SEM was applied to estimate the contribution of factors to PWB and to evaluate the moderating effect of CS on the relationship between EI and PWB. In the model, EI and PWB were added in step 1. Finally, EI, CS and PWB were added in step 2. The hypothesis regarding the moderating effect of the CS was supported if the interactions were significant. All statistical analyses performed with two‐tailed *p* <.01 were considered statistically significant.

## 4 RESULTS

### 4.1 Demographic participant characteristics

The demographic characteristics of the study subjects are shown in Table 1. As can be seen, more than half the participants held a bachelor's degree. The number of male participants (67.8%) is twice as much as that of female participants. The HCWs who have high PWB scores are holders of master's degrees and above (40.33 ± 5), aged 35–50 years (37.48 ± 4) and females (37.55 ± 5) in terms of the educational level, age and gender groups, respectively.

**TABLE 1:** Demographic characteristics (*N* = 146)

	Variables	*N*	%
Level of education	Associate degree	53	36.3
	Bachelor's degree	84	57.5
	Master's degree and above	9	6.2
Age	20–34 years	113	77.4
	35–50 years	23	15.8
	>50 years	10	6.8
Gender	Female	47	32.2
	Male	99	67.8

### 4.2 The correlations amongst emotional intelligence, CS and PWB

**FIGURE 1:**
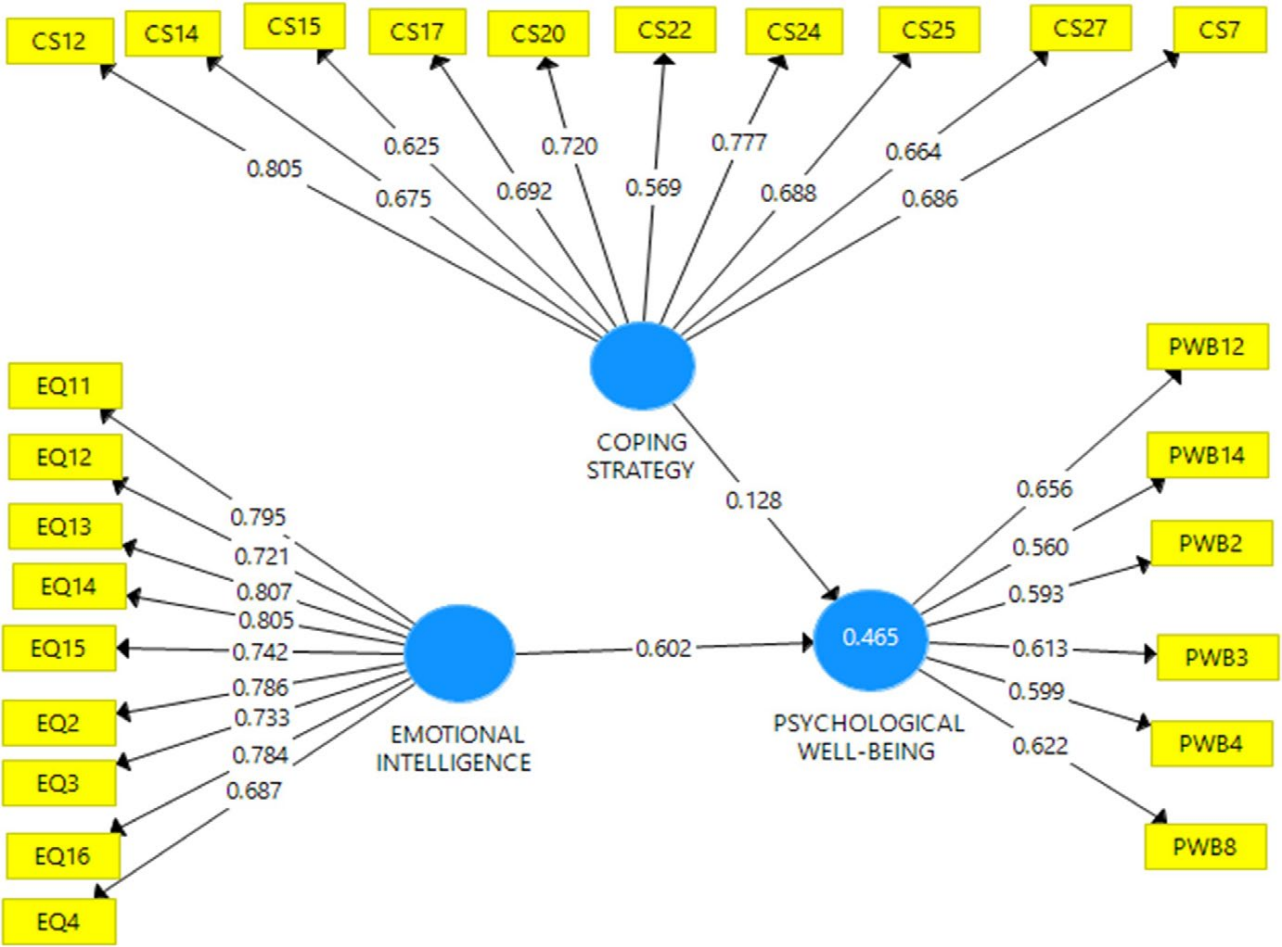
Structural model and measurement with loading values

**FIGURE 2:**
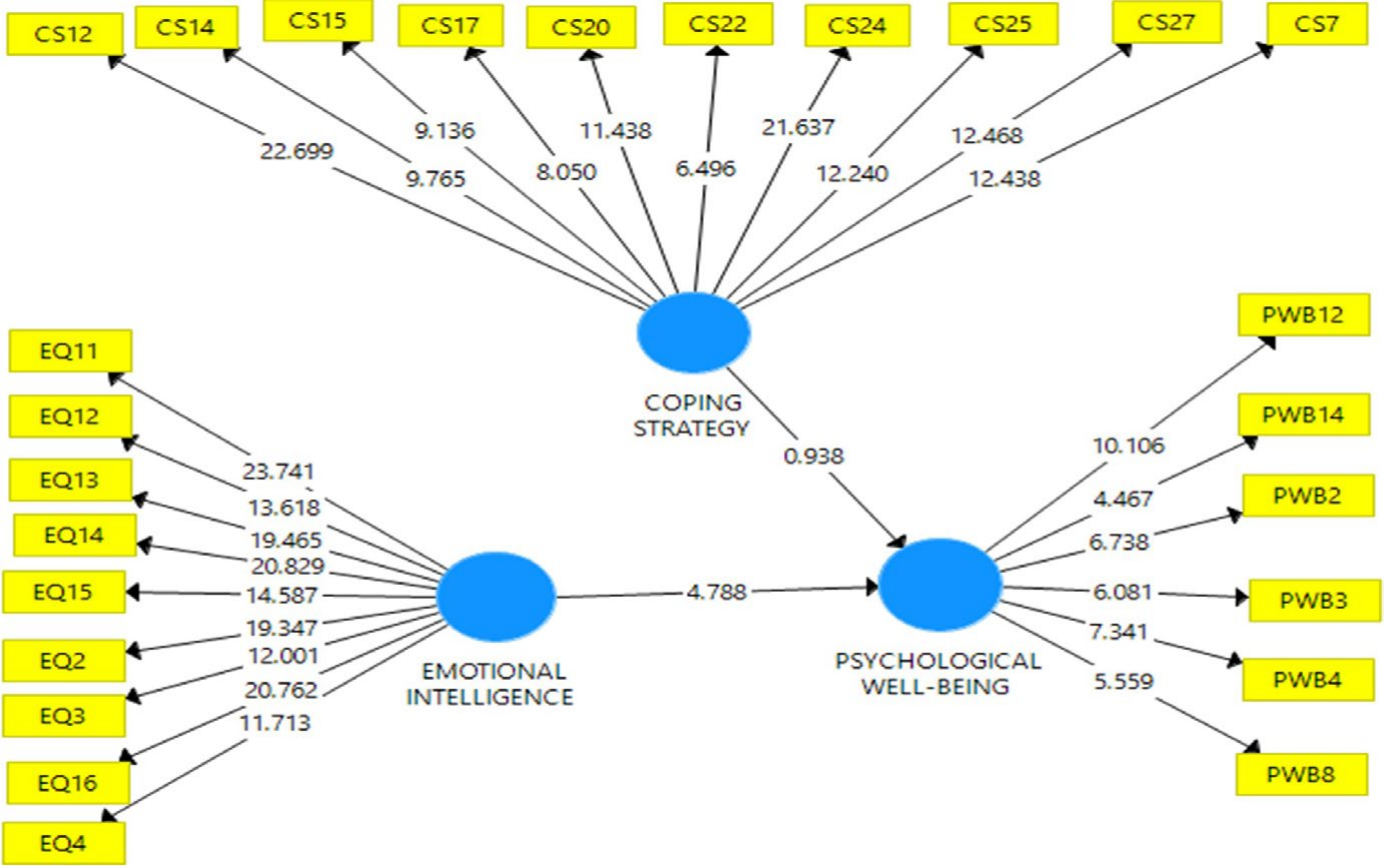
Structural model and measurement with *T* statistic

**FIGURE 3:**
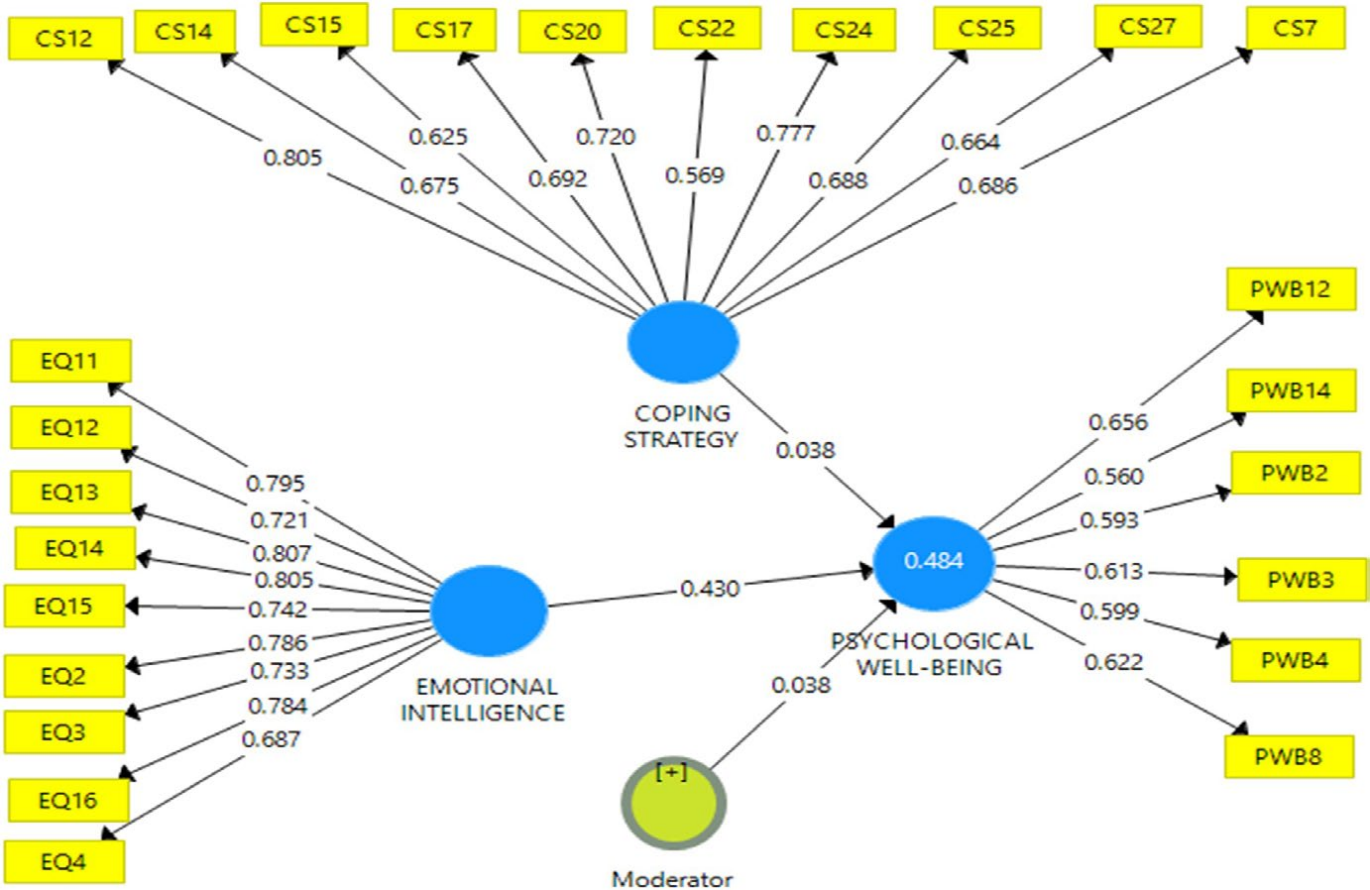
Structural model and measurement with moderator variables

**FIGURE 4:**
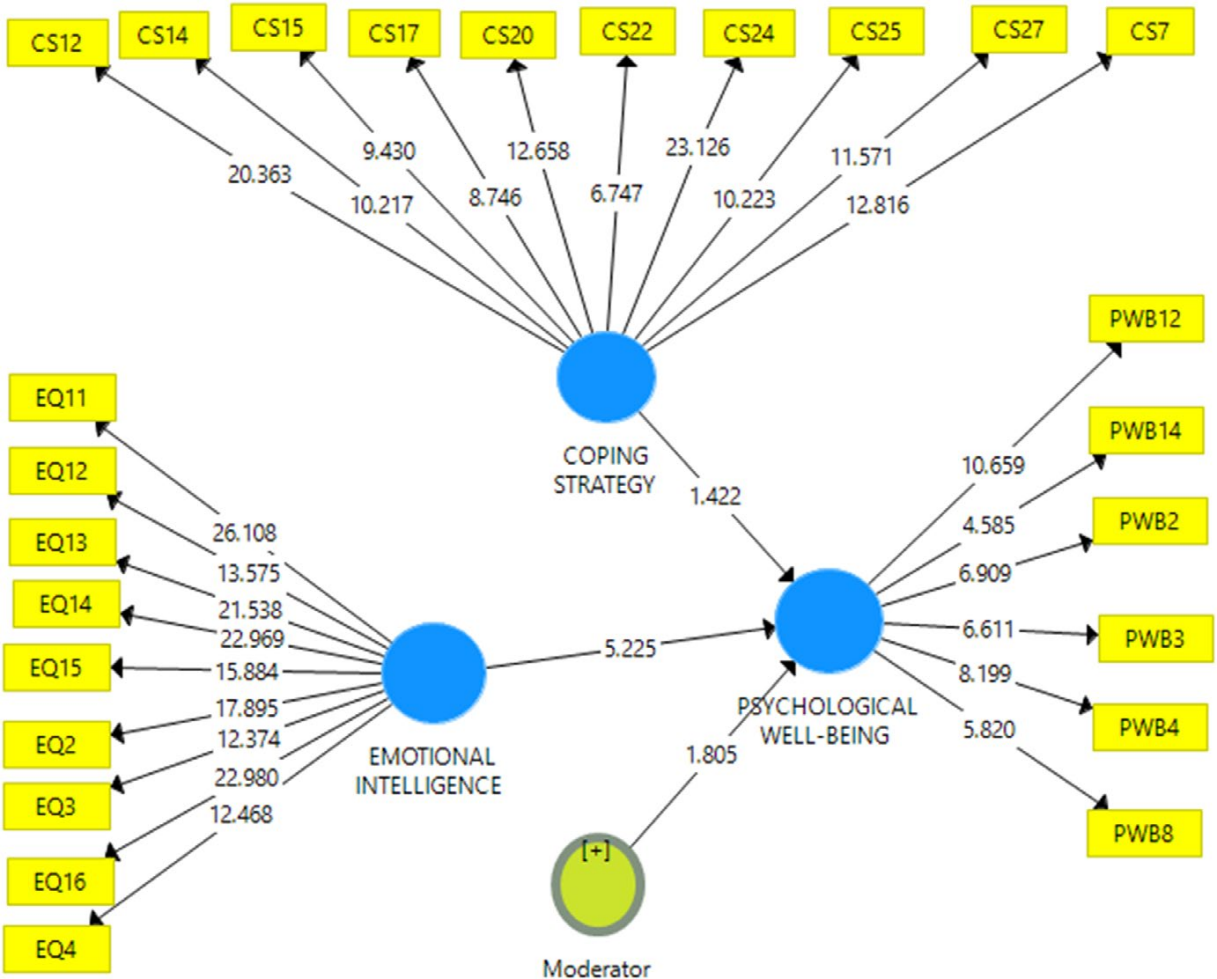
Structural model and measurement with moderator variables, the value of *T* statistic

Research data reveal that emotional intelligence has a significant effect on PWB (*t* = 5.225; *p* =.00), while CS has no significant effect on PWB (*T* = 1.422; *p* =.156). If a CS serves as a moderating variable, the relationship between emotional intelligence and PWB appears significant (*T* = 1.805; *p* =.072). This illustrates that CS is not a variable that directly affects PWB; rather, it is a variable that strengthens the relationship between emotional intelligence and PWB. This indicates that CS moderates the relationship between emotional intelligence and PWB in a positive direction. In other words, PWB increases if emotional intelligence is higher, especially if the CS is also high and vice versa (Table 2 and Figs 1, 2, 3, 4).

**TABLE 2:** The coefficient, standard deviation, *T* and *p* values of the latent variable relationship

Relationship path	Original sample (O)	Sample mean (M)	Standard deviation (STDEV)	*T* statistics (|O/STDEV|)	*p* values
Coping strategy → psychological well‐being	.175	.207	.123	1.422	.156
Emotional intelligence → psychological well‐being	.574	.553	.110	5.225	.000
Moderator → psychological well‐being	.092	.092	.051	1.805	.072

CS is a latent variable comprising valuable measuring items indicated by the loading value (in Table 3, Column 2) and is greater than the loading values of other latent variables. The loading value of the EI variable, shown in column (3), also indicates a correct measuring item and the PWB latent variable in column (5). The measurement model is considered valid when the cross‐loading value of each latent variable is greater than the loading values of other latent variables. In the reliability aspect in Table 4, it shows that the Cronbach's α value is greater than .600, the rho *A* value is more significant than .800, and the composite reliability is greater than .70, all indicating that the construction of latent variables in the measurement model is reliable. However, in this model, the R square value of the effects of CS and emotional intelligence is only .484 (Table 5).

**TABLE 3:** Latent variables for cross‐loading indicators

Indicator/Item code	Coping strategy	Emotional intelligence	Moderator	Psychological well‐being
(1)	(2)	(3)	(4)	(5)
CS12	.805	.509	−.216	.344
CS14	.675	.396	−.144	.275
CS15	.625	.296	−.183	.260
CS17	.692	.322	−.229	.307
CS20	.720	.429	−.085	.404
CS22	.569	.273	−.260	.270
CS24	.777	.388	−.091	.346
CS25	.688	.431	−.181	.381
CS27	.664	.372	−.091	.312
CS7	.686	.396	−.029	.242
Emotional intelligence * coping strategy	−.216	.011	1.000	.113
EQ11	.479	.795	−.009	.615
EQ12	.506	.721	−.056	.615
EQ13	.415	.807	.016	.475
EQ14	.358	.805	.082	.521
EQ15	.499	.742	−.060	.479
EQ16	.474	.784	−.060	.544
EQ2	.483	.786	.055	.460
EQ3	.272	.733	.067	.448
EQ4	.267	.687	.088	.381
PWB12	.430	.467	.003	.656
PWB14	.166	.342	.014	.560
PWB2	.270	.357	.235	.593
PWB3	.165	.449	−.005	.613
PWB4	.416	.468	.175	.599
PWB8	.134	.309	−.032	.622

**TABLE 4:** Latent variable reliability index

	Cronbach's Alpha	rho *A*	Composite reliability	Average variance extracted (AVE)
Coping strategy	.878	.886	.902	.480
Emotional intelligence	.911	.916	.926	.583
Moderator	1.000	1.000	1.000	1.000
Psychological well‐being	.666	.660	.778	.369

**TABLE 5:** *R* square‐dependent variable

	*R* square	*R* square adjusted
Psychological well‐being	.484	.473

## 5 DISCUSSION

PWB during the COVID‐19 pandemic is an important issue discussed in many studies. During this time, HCWs are one of the most vulnerable workgroups at risk of low PWB, because they face excessive workloads and work hours as they care for their patients (McDougall et al., 2021a).

A study conducted by Chen et al. (2020) discovered that HCWs often refused to receive psychological help, claiming that they have no difficulties, even though they reported feeling excited and irritable, lacking rest and other indicators of mental stress. It contradicts several other studies, which indicate that excessive work workload increases burnout symptoms, thus recommending that nursing staff should be encouraged to take sick leaves more regularly (Kowalczuk et al., 2020; de Oliveira et al., 2017). This confirms the need to search for other factors correlated with PWB. In this work, we hypothesized that emotional intelligence and CS positively correlate to PWB and that CS moderates the relationship between emotional intelligence and PWB.

The influence of emotional intelligent and CS on PWB amongst HCWs has not been analysed comprehensively so far. In the current study, the PLS analysis we developed allowed us to investigate the effects of emotional intelligence on PWB, with CS as a moderator. Based on the findings, we concluded that emotional intelligence has a positive association with PWB. In other words, emotional intelligence has an impact on HCWs’ PWB. Specifically, emotional intelligence appears to be a source of strength enabling HCWs to face various factors in their work that cause low PWB, such as excessive workload and threats to their mental and physical health (Kowalczuk et al., 2020; de Oliveira et al., 2017).

PWB is a sense of feeling well and having a complete understanding of one's life, including its spiritual dimensions (Burdorf et al., 2020). Ryff (2014) emphasized that individuals who have high levels of PWB are often characterized by self‐acceptance (positive attitude towards oneself), autonomy, environmental mastery, purpose in life, positive relationships with others and personal development. According to our results, higher values in emotional intelligence measures translate to increased PWB. Therefore, emotional intelligence capability predicts facets of personal well‐being and a positive relationship between life satisfaction and subjective happiness. Similarly, the study conducted by Di Fabio and Saklofske (2021); Malinauskas and Malinauskiene (2018) confirmed that emotional intelligence could be one of the elements that can promote happiness and PWB.

According to the three emotional intelligence indexes, a direct relationship between emotional intelligence and PWB has been developed: (a) knowing one's own emotions, (b) knowing other people's emotions and (c) regulating others' emotions. As the sense of PWB improves, participants understood others' emotions better and displayed an even better ability to understand their own emotions and control them (Antinienė & Lekavičienė, 2017).

On the one hand, in the integrated models, emotional intelligence is a compendium of stable personality characteristics, motivational aspects, socioemotional competencies and various cognitive abilities. On the other hand, the ability model defines “emotional intelligence” as an ability to process emotional knowledge (Salovey & Sluyter, 1997).

Another hypothesis presented in the current study is that CS has no significant effect on PWB, although it moderates the relationship between emotional intelligence and PWB. The results of the PLS‐SEM analysis we conducted revealed that CS is not a variable that directly affects PWB but is a variable that strengthens the relationship between emotional intelligence and PWB. In other words, the level of PWB increases if emotional intelligence is higher, especially if CS is also high, and vice versa. This finding is different from the findings of previous studies, which stated that positive coping correlates to higher PWB levels, while negative coping correlates to lower PWB levels (Gustems‐Carnicer et al., 2019; Meng & D'Arcy, 2016; Trucchia et al., 2013). A similar result confirmed by Philip and Cherian (2020) indicated that poor CS is one factor that influences the PWB of HCWs during an outbreak. Additionally, the coping strategies of denial and problem‐solving have been identified as significant predictors of well‐being. Additionally, the coping strategies of denial and problem‐solving have been identified as significant predictors of well‐being in schizophrenia studies (Rammohan et al., 2002). Finally, coping strategies have been shown to influence the PWB of gastric cancer patients (Krok & Telka, 2019).

In line with a study of Por et al. (2011), our analysis indicated that improved feelings of control and emotional intelligence enable nursing students to deal with stress, allowing them to develop active and successful coping strategies, which in turn increase their subjective well‐being. Furthermore, a study by Sygit‐Kowalkowska et al. (2015) found that a higher level of declared active coping caused a higher level of mental well‐being. In the current study, we found that the significant predictors of PWB included sense of humour, seeking support, and levels of emotional control.

Furthermore, we also found that the moderating effect of CS may be explained by the fact that emotional intelligence is a skill that maximizes PWB. Some authors have identified correlations amongst emotional intelligence, coping strategies and mental well‐being (Gerits et al., 2005). For example, Pau and Croucher (2003) found that, on the one hand, less perceived stress and better health and well‐being were experienced by individuals with high emotional intelligence. Those with low emotional intelligence, on the other hand, had inadequate coping capacity and, therefore, felt more unhappy.

The current study implies that HCWs can increase their PWB by increasing positive relationships with others in accordance with emotional intelligence indicators, such as maintaining an excellent patient–HCW relationship. Similarly, one study revealed that coping strategies and emotional intelligence can be achieved by improving health behaviours and physical self‐esteem in sports activities during the COVID‐19 pandemic (Edwards et al., 2004, 2005). Healthy behaviour and exercise can help HCWs increase their immunity and affect their ability to solve problems at work, which in turn, improves their PWB.

## 6 STRENGTH AND LIMITATIONS

The strength of this study is that it uses a sample from clinics, community health centre, and hospitals from several major cities in Indonesia that serve COVID‐19 patients. Thus, the data obtained genuinely represent the conditions currently faced by Indonesian HCWs. Furthermore, this is national study aiming to assess the PWB of HCWs in Indonesia. There is an important message in the Indonesian context related to providing better support and improving HCWs' working conditions to increase their PWB. Understanding the factors contributing to their emotional intelligence and CS will help increase their well‐being.

There are several limitations to this study. First, the collected data were obtained using self‐reported procedures. Second, it may also involve some bias in terms of social desirability. Thus, we tried to reduce the possibility of this bias by reminding the participants about the confidentiality of their replies. Furthermore, causality could not be determined due to the cross‐sectional design of this study. Thus, future studies are recommended to undertake longitudinal studies with multi‐level framework measurements.

## 7 CONCLUSIONS

We conclude that CS moderates the correlation between emotional intelligence and PWB. Our findings highlight the significance of providing timely and effective intervention in these variables for the improvement of PWB. Developing emotional intelligence and coming up with coping strategies are crucial strategies to improve the PWB of HCWs, especially during the COVID‐19 pandemic.

## CONFLICT OF INTEREST

The authors declare that there is no conflict of interest that could be perceived as prejudicing the impartiality of the research reported.

## RESEARCH ETHICS COMMITTEE APPROVAL

This protocol was accepted by health research ethics committee University of Muhammadiyah Malang No. “E.5.a/038/KEPK‐UMM/III/2021.” All participants were fully informed about the objectives of this study and were assured that all the information they provided would only be used for the study. All respondents signed informed consent forms before the completion of the questionnaire.

## Data Availability

The data supporting the results of this study are available from the corresponding author upon request.
